# Sintilimab combined with chemotherapy in advanced pulmonary epithelioid hemangioendothelioma: a case report and translational insights

**DOI:** 10.3389/fonc.2025.1575039

**Published:** 2025-11-20

**Authors:** Lijun Jing, Huiyun An, Hao Yang, Liming Zhang, Tongyuan Li, Yongming Wang

**Affiliations:** 1 Department of Anesthesiology, Weifang No.2 People’s Hospital, Weifang, Shandong, China; 2 Department of Thoracic Surgery, Weifang No.2 People’s Hospital, Weifang, Shandong, China

**Keywords:** pulmonary epithelioid hemangioendothelioma, sintilimab, PD-L1, immunotherapy, WWTR1-CAMTA1 fusion

## Abstract

**Background:**

Pulmonary epithelioid hemangioendothelioma (PEH) is an exceedingly rare vascular tumor, presenting a significant challenge due to its limited treatment options. Immunotherapy in combination with chemotherapy emerges as a potential frontier, yet the understanding of its application in PEH remains in its infancy.

**Methods:**

A male patient initially faced misdiagnosis as having aspergillosis. Through histopathology and immunohistochemistry, a definitive diagnosis of PEH was later established. The treatment journey involved surgical resection, followed by chemotherapy with albumin-bound paclitaxel and carboplatin, and finally immunotherapy with sintilimab.

**Results:**

A remarkable radiological improvement was observed post-sintilimab administration, leading to disease stabilization. Significantly, this is the first-ever report of the efficacy of sintilimab in PD-L1-high PEH, filling a critical gap in the existing literature.

**Conclusion:**

This case not only underscores the potential of sintilimab in PD-L1-high PEH but also sets a precedent for further exploration of immune checkpoint inhibitors in this rare disease.

## 1 Case introduction

### 1.1 Initial presentation and misdiagnosis

In June 2024, a 63-year-old male patient visited Weifang People’s Hospital due to “cough and hemoptysis for 1 month”. Chest CT examination showed an inflammatory lesion in the lower lobe of the right lung. The imaging features presented as a large patchy high-density shadow in the right lower lobe, with uneven density and blurred edges, highly suggestive of an inflammatory condition. Bronchoscopic biopsy was performed, and the result showed visible hyphae, leading to a diagnosis of *Aspergillus* infection. However, the patient did not undergo other diagnostic tests to confirm or exclude aspergillosis. (1) He was then given oral “voriconazole tablets” for treatment. As early as April 2024, the patient had visited another hospital prior due to “hemoptysis” and underwent vascular interventional treatment, but the surgical effect was poor, and intermittent hemoptysis still occurred.

### 1.2 Definitive diagnosis

**Figure 1:**
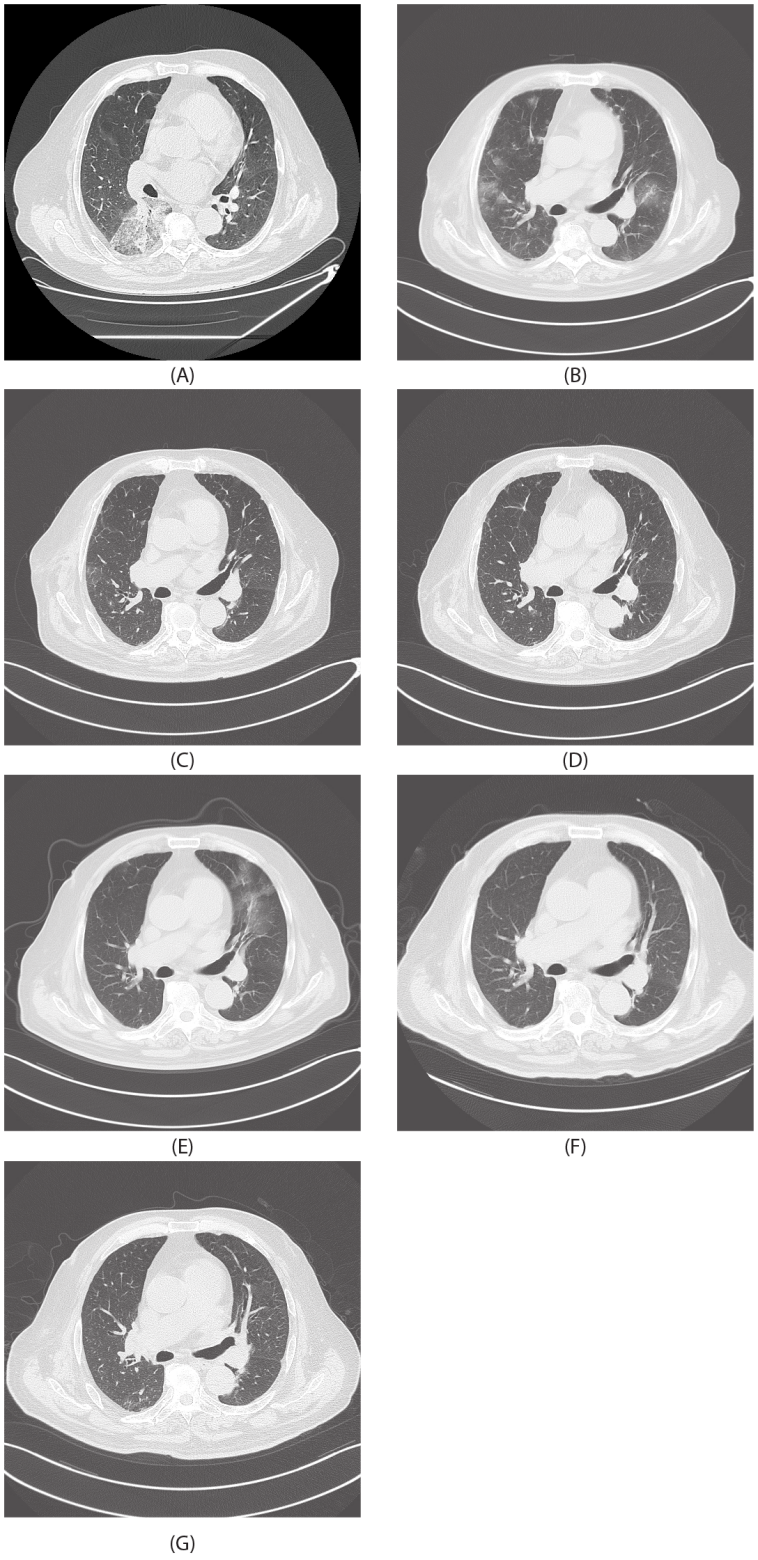
Radiological images. **(A)** July 30, 2024. **(B)** August 20, 2024. **(C)** September 11, 2024. **(D)** October 11, 2024. **(E)** November 13, 2024. **(F)** December 13, 2024. **(G)** January 12, 2025.

**Figure 2:**
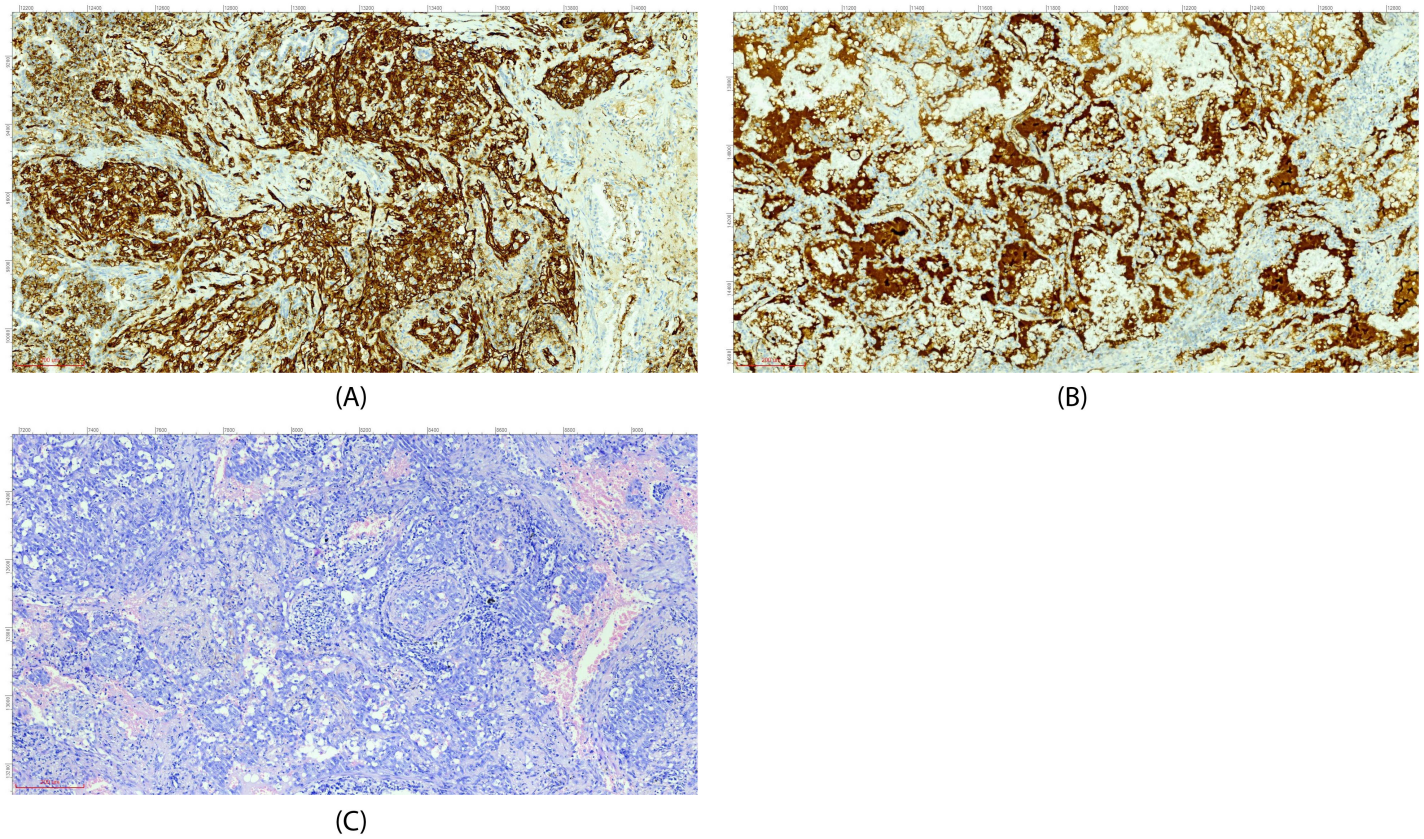
Immunohistochemical staining (**A**, CD31; **B**, Factor VIII) and H&E morphology **(C)** (all images at ×200 magnification).

On July 25, 2024, the patient was first admitted to our hospital. Chest CT showed consolidation and ground-glass shadows in the lower lobe of the right lung with unclear boundaries, and uniform enhancement on enhanced scanning (**Figure 1A**). After performing additional examinations and preparing the patient for surgery, video-assisted thoracoscopic resection of the dorsal segment of the lower lobe of the right lung was performed on July 30, 2024. The postoperative pathological biopsy report indicated a mesenchymal-derived malignant tumor, consistent with epithelioid hemangioendothelioma. The immunohistochemical results were as follows: CK (weak+), CK7 (−), Napsin A (−), TTF−1 (−), Vimentin (+), P40 (−), Ki-67 (accounting for approximately 70%), Factor VIII (+), CD31 (+), and HMB-45 (−) (**Figure 2**) According to the diagnostic criteria in literature, the positive expression of vascular endothelial markers like CD31 and Factor VIII and the specific morphological features of tumor cells under the microscope are crucial for the diagnosis of pulmonary epithelioid hemangioendothelioma (PEH) (2).

### 1.3 Follow-up and further treatments

On August 20, 2024, the patient had another episode of hemoptysis. A chest CT scan revealed diffuse changes in both lungs (**Figure 1B**). After a comprehensive analysis of the clinical condition, the diagnosis was progression of the disease. The patient was given albumin-bound paclitaxel 400 mg d1 combined with carboplatin 400 mg d1 for chemotherapy, along with tropisetron and dexamethasone to reduce chemotherapy adverse reactions, and then discharged for convalescence.

On September 11, 2024, the patient was re-admitted. Chest spiral CT showed that the diffuse lesions in both lungs were partially absorbed compared to those on August 20, 2024 (**Figures 1B, C**). The chemotherapy regimen of albumin-bound paclitaxel 400 mg d1 combined with carboplatin 400 mg d1 was continued for two more cycles.

On November 13, 2024, chest spiral CT showed that after partial resection of the right lower lobe of the lung, there were diffuse ground-glass lesions in both lungs, which were partially enlarged compared to those shown on the chest CT on October 11, 2024 (**Figures 1D, E**). The patient had hemoptysis again, suggesting that the efficacy of albumin-bound paclitaxel was poor. Pathological PD-L1 testing showed 40%, and tumor next-generation sequencing (NGS) testing (3) showed no mutations. The treatment was changed to sintilimab immunotherapy combined with gemcitabine 1.8 g d1 and d8 + carboplatin 400 mg d1 chemotherapy, along with sintilimab 200 mg immunotherapy. On December 13, 2024, a chest CT review showed that the lesions in both lungs had improved significantly (**Figure 1F**). Chemotherapy combined with immunotherapy was continued, and the patient’s condition is currently stable (**Figure 1G**).

## 2 Discussion

### 2.1 Diagnosis and differential diagnosis

PEH is a rare angiosarcoma originating from vascular endothelial cells. Its pathological features are of crucial significance in diagnosis. Under the microscope, the tumor cells show an epithelioid morphology, mostly polygonal or round, with abundant eosinophilic cytoplasm, large and irregular nuclei, and visible nucleoli; some cells also have vacuoles, which can squeeze the nucleus to one side, presenting a signet ring-like appearance. The tumor cells are often arranged in cord-like, nest-like, or gland-like structures, with thin fibrovascular stroma interspersed. Characteristic eosinophilic hyaline material deposition can also be seen in the tumor, which is round or oval, positive for Periodic Acid-Schiff (PAS) staining, and resistant to amylase digestion (4). In addition, the tumor cells can invade blood vessels and lymphatic vessels and form tumor thrombi in blood vessels or lymphatic vessels. Its clinical symptoms are non-specific, often manifested as cough, hemoptysis, chest pain, etc., similar to other lung diseases (5). As in this case, it was initially misdiagnosed as a fungal infection. In this section, we will focus more on the aspects related to immunotherapy rather than repeating the detailed pathological features described in the case introduction. On imaging, PEH mostly presents as multiple nodules or masses in both lungs, which may be accompanied by ground-glass shadows, consolidation shadows, etc., and is easily confused with lung metastases, lung cancer, inflammatory lesions, etc. Pathological examination and immunohistochemistry are the keys to diagnosis. Positive expression of vascular endothelial markers such as CD31, CD34, and Factor VIII, and negative or weakly positive expression of epithelial markers such as CK and TTF-1 in immunohistochemistry, are helpful for differential diagnosis.

### 2.2 Treatment principles

The treatment principles of PEH are comprehensively considered according to the tumor stage, the patient’s physical condition, etc. For early-stage limited lesions, surgical resection is the main treatment method and can achieve a radical cure (6). However, most patients have advanced-stage diseases at the time of diagnosis and lose the opportunity for surgery. At this time, comprehensive treatment methods such as chemotherapy, targeted therapy, and immunotherapy have become the main choices (7). Chemotherapy regimens mostly use platinum-based drugs combined with other drugs. For example, the albumin-bound paclitaxel combined with a carboplatin chemotherapy regimen was used in this case, but some patients may be insensitive to chemotherapy. For example, the efficacy of albumin-bound paclitaxel was poor in this case.

### 2.3 Treatment status of immunotherapy in this disease

In recent years, immunotherapy has made significant progress in a variety of malignant tumors. Immune checkpoint inhibitors can block the immune checkpoint and activate the body’s own immune system to kill tumor cells (8). For PEH, immunotherapy is still in the exploratory stage.

In this case, the pathological PD-L1 test showed 40%, and no mutations were detected by tumor NGS. No mutations, including single-nucleotide variants (SNVs), copy number variations (CNVs), and gene fusions, were detected. The negative NGS result indicates that there are no common actionable mutations in this patient, highlighting the potential role of immunotherapy.

Notably, this is the first report of a PD-1 inhibitor (sintilimab) combined with chemotherapy in an Asian population with PD-L1-high PEH. This finding represents a significant leap forward in the field, as it provides the first-hand evidence of the potential efficacy of sintilimab in this specific subset of patients.

Studies have shown that the expression level of PD-L1 may be related to the efficacy of immunotherapy, and patients with high expression may benefit more from immunotherapy (9). In vascular-derived tumors, high PD-L1 expression may be associated with an immunosuppressive microenvironment. For example, in a study on angiosarcoma (PMID 33472621), high PD-L1 expression was found to be related to the infiltration of immunosuppressive cells such as regulatory T cells, which may also be the case in PEH.

There are also many achievements in the immunotherapy research of other lung cancer types, which can provide references for the immunotherapy of pulmonary epithelioid hemangioendothelioma (10–14).

In recent years, studies have found that approximately 90% of PEH cases have characteristic gene fusion events, among which WWTR1–CAMTA1 fusion is the most common molecular driver event. This fusion may be related to the immune-evasion mechanism of the tumor (15). Understanding these molecular features may help predict the sensitivity of PEH to immunotherapy, but more research is needed in this area.

In terms of safety, the patient did not experience any immune-related adverse events (irAEs). No immune-related pneumonia was observed, nor were there any thyroid function-related irAEs detected during the treatment period.

## 3 Conclusion

Pulmonary epithelioid hemangioendothelioma is clinically rare, difficult to diagnose, and easy to misdiagnose. Treatment needs to comprehensively consider various factors. Immunotherapy combined with chemotherapy provides a new treatment option for some patients. This case, as the first report of sintilimab’s efficacy in PD-L1-high PEH, highlights the potential of immune checkpoint inhibitors in selected vascular sarcoma subtypes. However, more high-quality clinical studies are still needed to clarify its efficacy and safety.

## Data Availability

The raw data supporting the conclusions of this article will be made available by the authors, without undue reservation.
